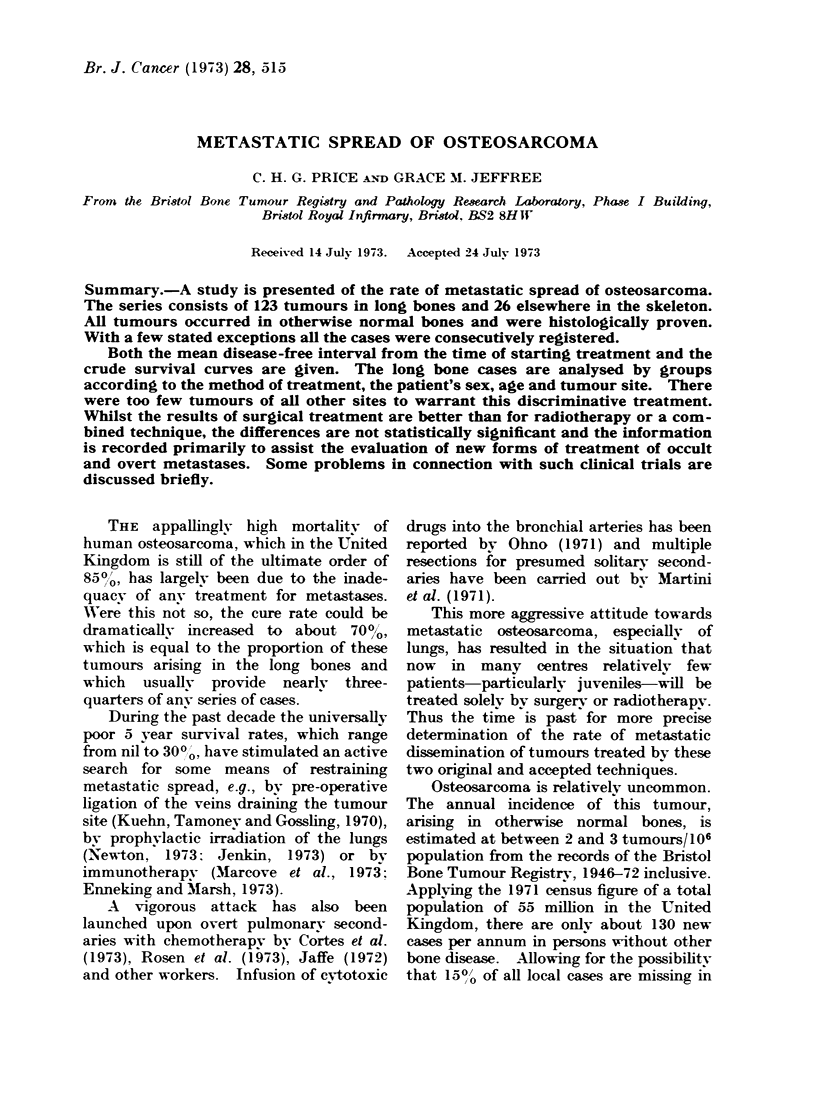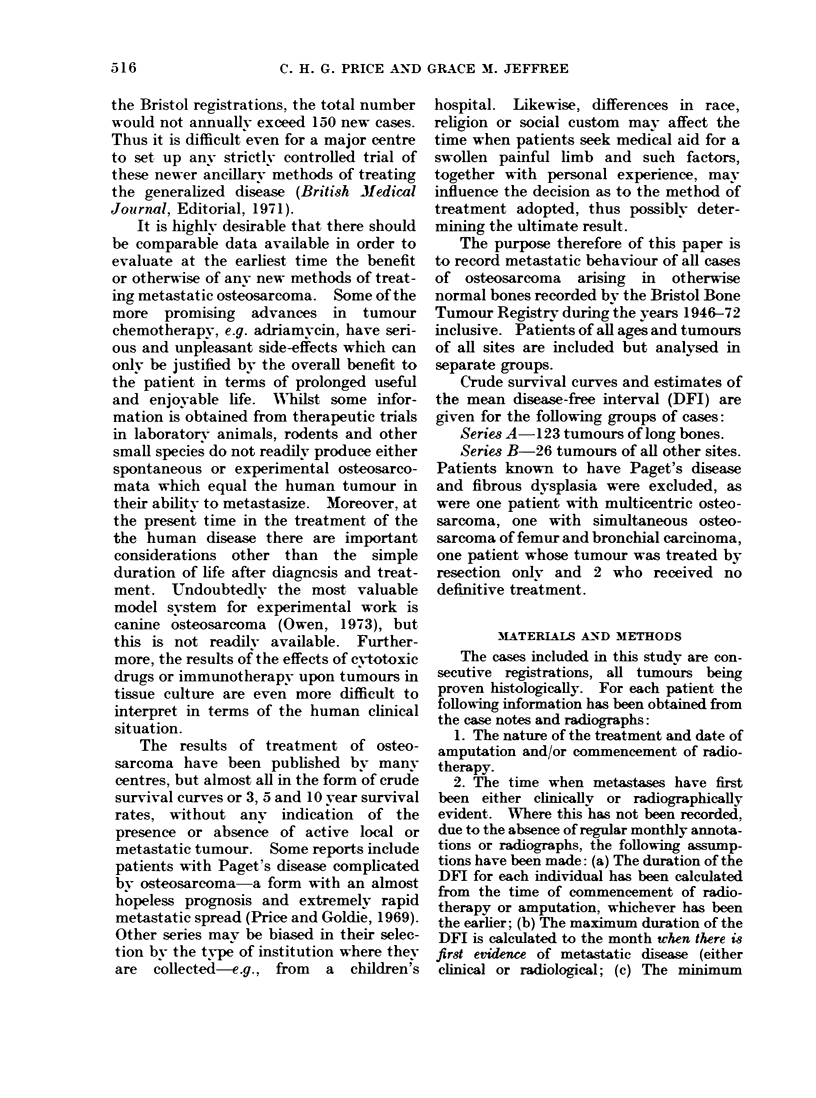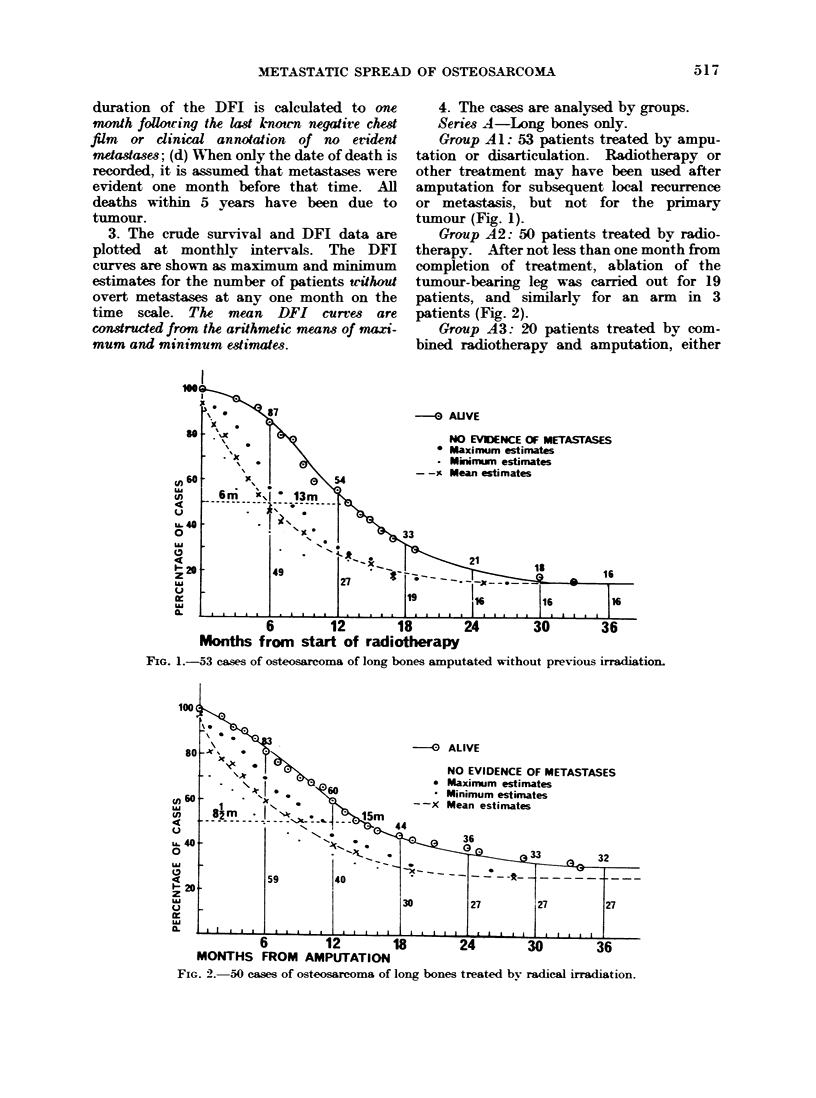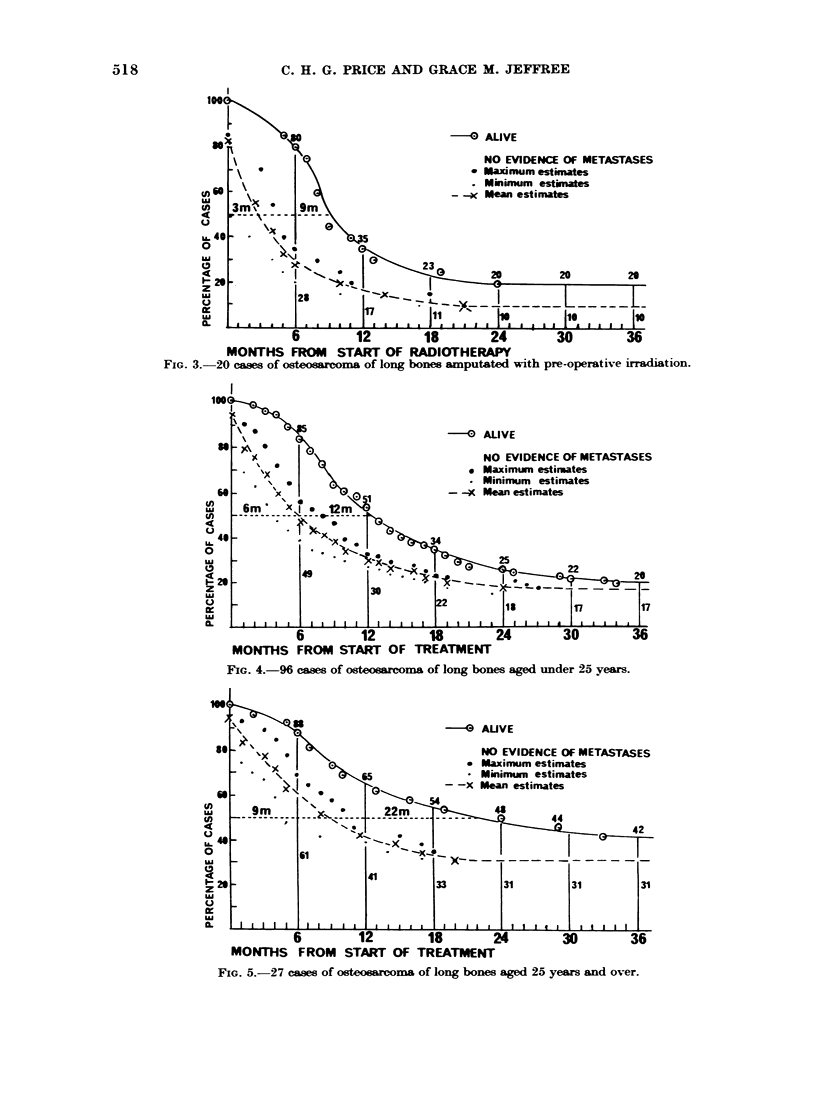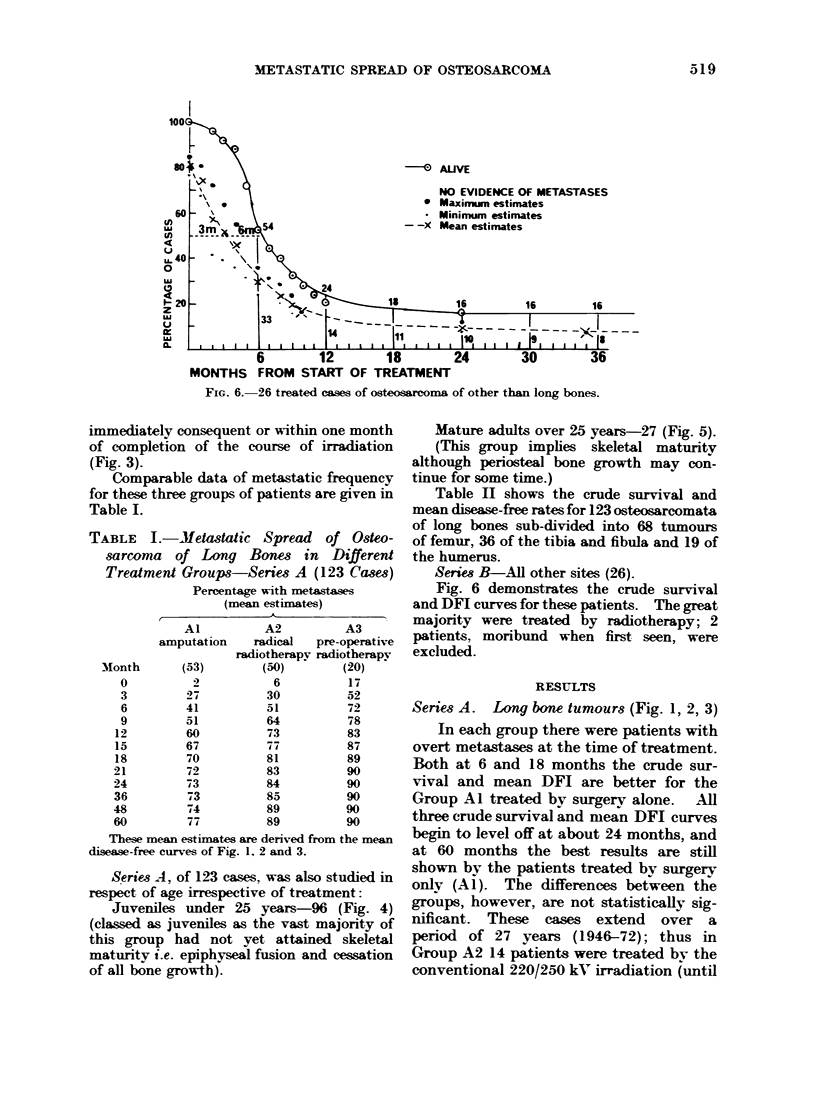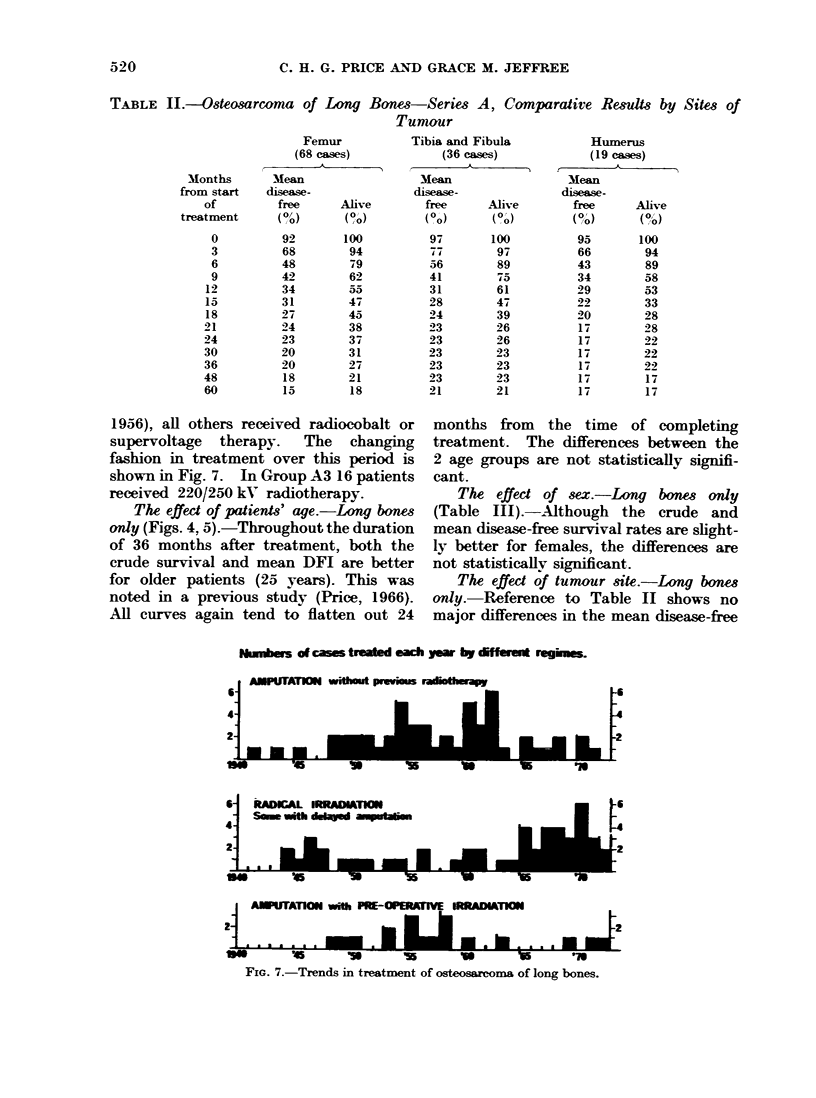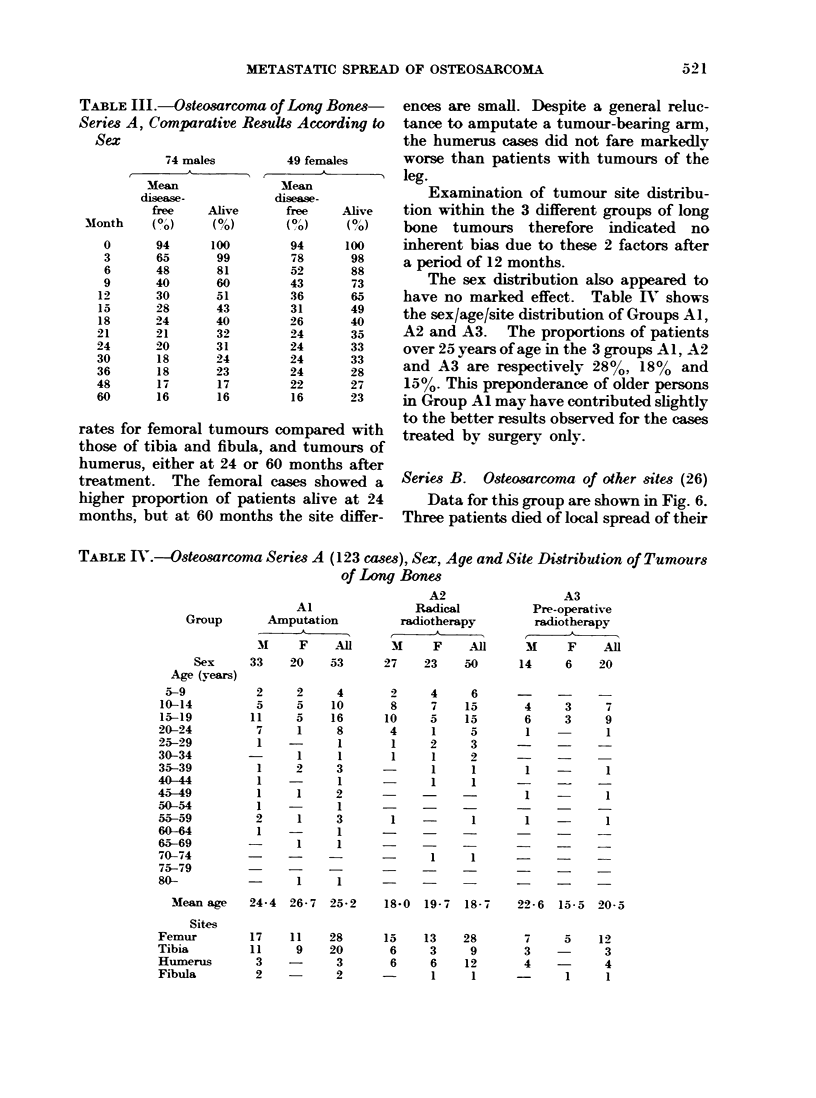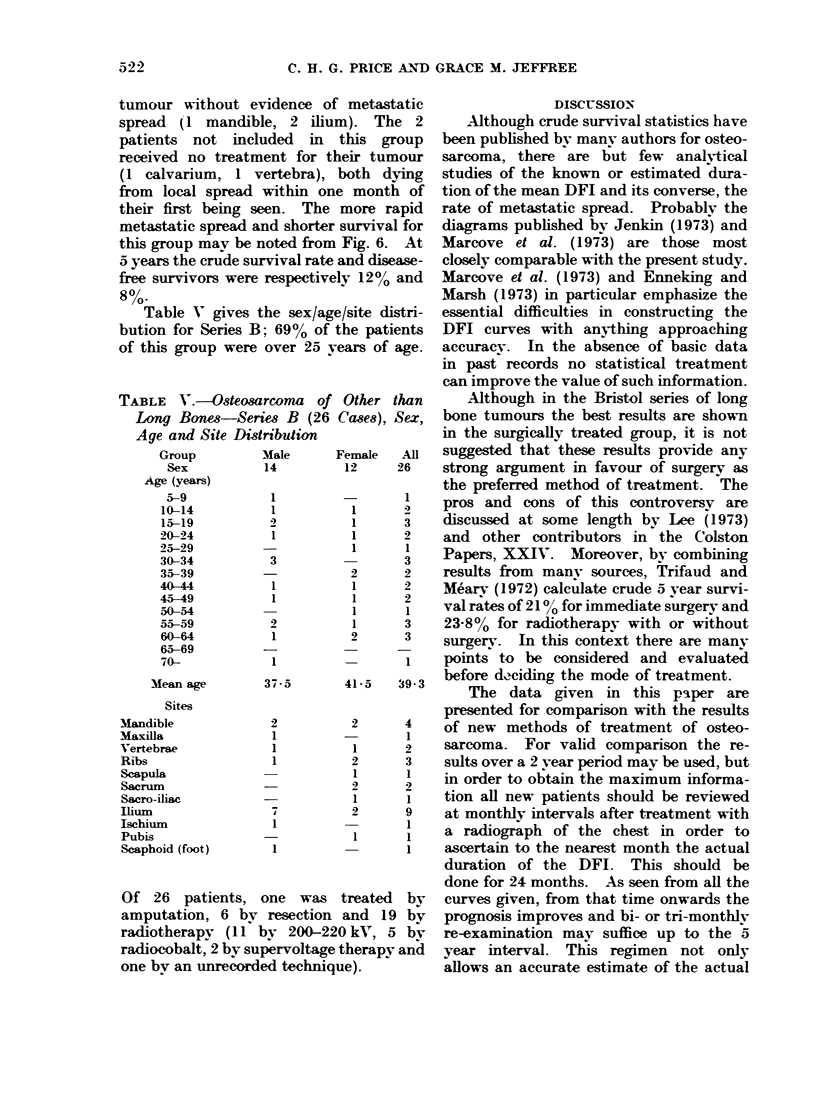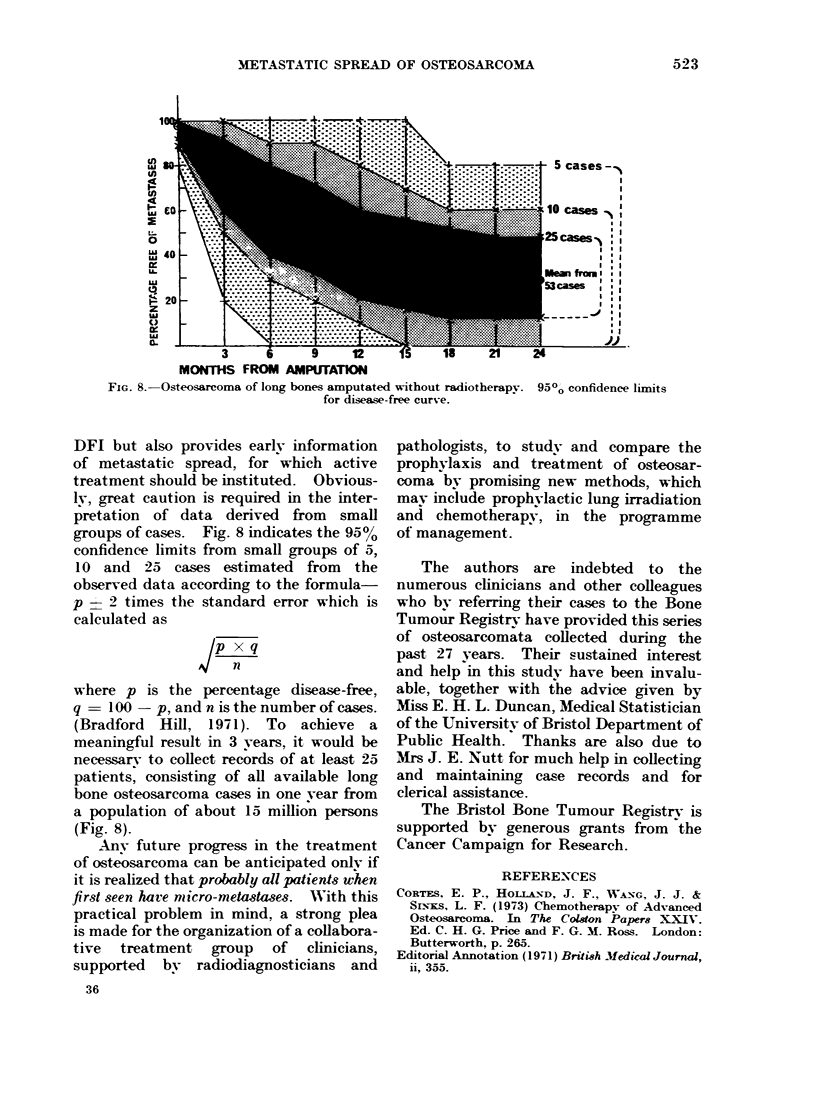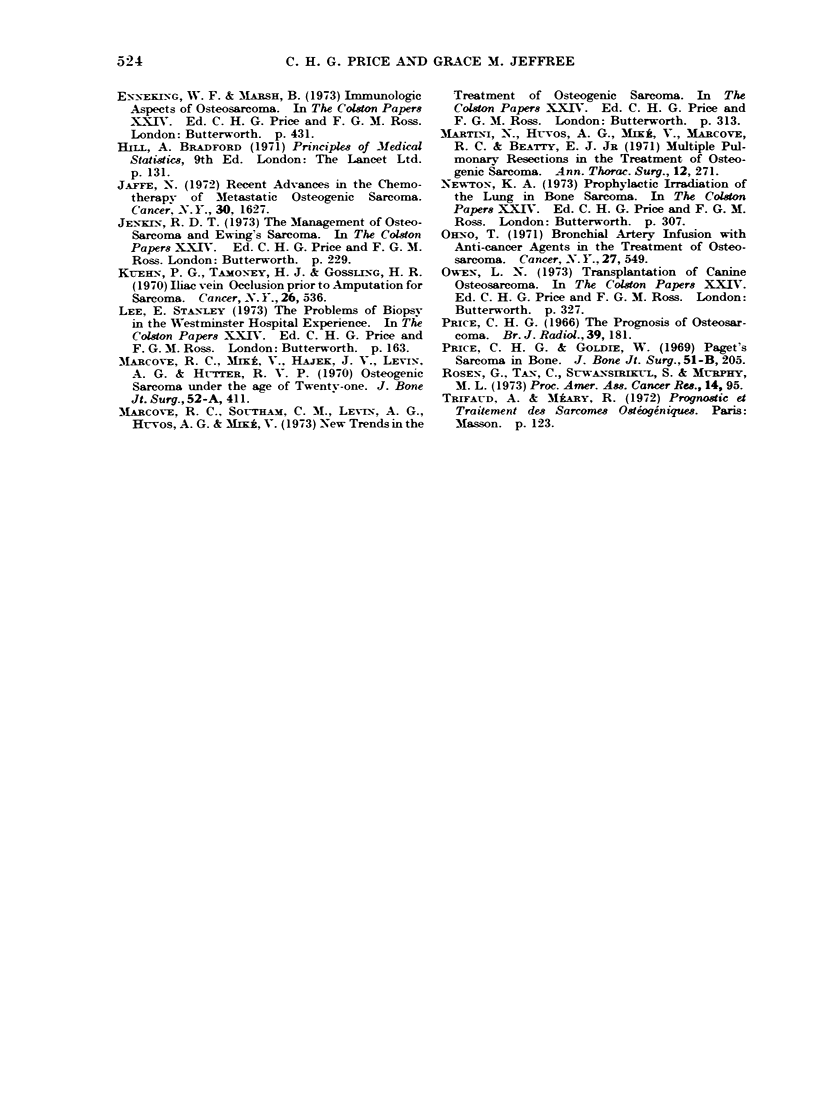# Metastatic Spread of Osteosarcoma

**DOI:** 10.1038/bjc.1973.181

**Published:** 1973-12

**Authors:** C. H. G. Price, Grace M. Jeffree

## Abstract

A study is presented of the rate of metastatic spread of osteosarcoma. The series consists of 123 tumours in long bones and 26 elsewhere in the skeleton. All tumours occurred in otherwise normal bones and were histologically proven. With a few stated exceptions all the cases were consecutively registered.

Both the mean disease-free interval from the time of starting treatment and the crude survival curves are given. The long bone cases are analysed by groups according to the method of treatment, the patient's sex, age and tumour site. There were too few tumours of all other sites to warrant this discriminative treatment. Whilst the results of surgical treatment are better than for radiotherapy or a combined technique, the differences are not statistically significant and the information is recorded primarily to assist the evaluation of new forms of treatment of occult and overt metastases. Some problems in connection with such clinical trials are discussed briefly.


*Br. J. Cancer* (1973) **28**, 515

### METASTATIC SPREAD OF OSTEOSARCOMA

C. H. G. PRICE AND GRACE M. JEFFREE

*From the Bristol Bone Tumour Registry and Pathology Research Laboratory, Phase I Building, Bristol Royal Infirmary, Bristol, BS2 8HW*

Received 14 July 1973. Accepted 24 July 1973

**Summary.—A study is presented of the rate of metastatic spread of osteosarcoma. The series consists of 123 tumours in long bones and 26 elsewhere in the skeleton. All tumours occurred in otherwise normal bones and were histologically proven. With a few stated exceptions all the cases were consecutively registered.**

**Both the mean disease-free interval from the time of starting treatment and the crude survival curves are given. The long bone cases are analysed by groups according to the method of treatment, the patient's sex, age and tumour site. There were too few tumours of all other sites to warrant this discriminative treatment. Whilst the results of surgical treatment are better than for radiotherapy or a combined technique, the differences are not statistically significant and the information is recorded primarily to assist the evaluation of new forms of treatment of occult and overt metastases. Some problems in connection with such clinical trials are discussed briefly.**

THE appallingly high mortality of human osteosarcoma, which in the United Kingdom is still of the ultimate order of 85%, has largely been due to the inadequacy of any treatment for metastases. Were this not so, the cure rate could be dramatically increased to about 70%, which is equal to the proportion of these tumours arising in the long bones and which usually provide nearly three-quarters of any series of cases.

During the past decade the universally poor 5 year survival rates, which range from nil to 30%, have stimulated an active search for some means of restraining metastatic spread, *e.g.*, by pre-operative ligation of the veins draining the tumour site (Kuehn, Tamoney and Gossling, 1970), by prophylactic irradiation of the lungs (Newton, 1973; Jenkin, 1973) or by immunotherapy (Marcove *et al.*, 1973; Enneking and Marsh, 1973).

A vigorous attack has also been launched upon overt pulmonary secondaries with chemotherapy by Cortes *et al.* (1973), Rosen *et al.* (1973), Jaffe (1972) and other workers. Infusion of cytotoxic drugs into the bronchial arteries has been reported by Ohno (1971) and multiple resections for presumed solitary secondaries have been carried out by Martini *et al.* (1971).

This more aggressive attitude towards metastatic osteosarcoma, especially of lungs, has resulted in the situation that now in many centres relatively few patients—particularly juveniles—will be treated solely by surgery or radiotherapy. Thus the time is past for more precise determination of the rate of metastatic dissemination of tumours treated by these two original and accepted techniques.

Osteosarcoma is relatively uncommon. The annual incidence of this tumour, arising in otherwise normal bones, is estimated at between 2 and 3 tumours/$10^6$ population from the records of the Bristol Bone Tumour Registry, 1946–72 inclusive. Applying the 1971 census figure of a total population of 55 million in the United Kingdom, there are only about 130 new cases per annum in persons without other bone disease. Allowing for the possibility that 15% of all local cases are missing in the Bristol registrations, the total number would not annually exceed 150 new cases. Thus it is difficult even for a major centre to set up any strictly controlled trial of these newer ancillary methods of treating the generalized disease (*British Medical Journal*, Editorial, 1971).

It is highly desirable that there should be comparable data available in order to evaluate at the earliest time the benefit or otherwise of any new methods of treating metastatic osteosarcoma. Some of the more promising advances in tumour chemotherapy, *e.g.* adriamycin, have serious and unpleasant side-effects which can only be justified by the overall benefit to the patient in terms of prolonged useful and enjoyable life. Whilst some information is obtained from therapeutic trials in laboratory animals, rodents and other small species do not readily produce either spontaneous or experimental osteosarcomata which equal the human tumour in their ability to metastasize. Moreover, at the present time in the treatment of the the human disease there are important considerations other than the simple duration of life after diagnosis and treatment. Undoubtedly the most valuable model system for experimental work is canine osteosarcoma (Owen, 1973), but this is not readily available. Furthermore, the results of the effects of cytotoxic drugs or immunotherapy upon tumours in tissue culture are even more difficult to interpret in terms of the human clinical situation.

The results of treatment of osteosarcoma have been published by many centres, but almost all in the form of crude survival curves or 3, 5 and 10 year survival rates, without any indication of the presence or absence of active local or metastatic tumour. Some reports include patients with Paget's disease complicated by osteosarcoma—a form with an almost hopeless prognosis and extremely rapid metastatic spread (Price and Goldie, 1969). Other series may be biased in their selection by the type of institution where they are collected—*e.g.*, from a children's hospital. Likewise, differences in race, religion or social custom may affect the time when patients seek medical aid for a swollen painful limb and such factors, together with personal experience, may influence the decision as to the method of treatment adopted, thus possibly determining the ultimate result.

The purpose therefore of this paper is to record metastatic behaviour of all cases of osteosarcoma arising in otherwise normal bones recorded by the Bristol Bone Tumour Registry during the years 1946–72 inclusive. Patients of all ages and tumours of all sites are included but analysed in separate groups.

Crude survival curves and estimates of the mean disease-free interval (DFI) are given for the following groups of cases:

*Series A*—123 tumours of long bones.

*Series B*—26 tumours of all other sites. Patients known to have Paget's disease and fibrous dysplasia were excluded, as were one patient with multicentric osteosarcoma, one with simultaneous osteosarcoma of femur and bronchial carcinoma, one patient whose tumour was treated by resection only and 2 who received no definitive treatment.

### MATERIALS AND METHODS

The cases included in this study are consecutive registrations, all tumours being proven histologically. For each patient the following information has been obtained from the case notes and radiographs:

1. The nature of the treatment and date of amputation and/or commencement of radiotherapy.

2. The time when metastases have first been either clinically or radiographically evident. Where this has not been recorded, due to the absence of regular monthly annotations or radiographs, the following assumptions have been made: (a) The duration of the DFI for each individual has been calculated from the time of commencement of radiotherapy or amputation, whichever has been the earlier; (b) The maximum duration of the DFI is calculated to the month *when there is first evidence* of metastatic disease (either clinical or radiological; (c) The minimum duration of the DFI is calculated to *one month following the last known negative chest film or clinical annotation of no evident metastases*; (d) When only the date of death is recorded, it is assumed that metastases were evident one month before that time. All deaths within 5 years have been due to tumour.

3. The crude survival and DFI data are plotted at monthly intervals. The DFI curves are shown as maximum and minimum estimates for the number of patients *without* overt metastases at any one month on the time scale. *The mean DFI curves are constructed from the arithmetic means of maximum and minimum estimates.*

4. The cases are analysed by groups.

*Series A*—Long bones only.

*Group A1:* 53 patients treated by amputation or disarticulation. Radiotherapy or other treatment may have been used after amputation for subsequent local recurrence or metastasis, but not for the primary tumour (Fig. 1).

*Group A2:* 50 patients treated by radiotherapy. After not less than one month from completion of treatment, ablation of the tumour-bearing leg was carried out for 19 patients, and similarly for an arm in 3 patients (Fig. 2).

*Group A3:* 20 patients treated by combined radiotherapy and amputation, either

FIG. 1.—53 cases of osteosarcoma of long bones amputated without previous irradiation.

FIG. 2.—50 cases of osteosarcoma of long bones treated by radical irradiation.

Fig. 3.—20 cases of osteosarcoma of long bones amputated with pre-operative irradiation.

Fig. 4.—96 cases of osteosarcoma of long bones aged under 25 years.

Fig. 5.—27 cases of osteosarcoma of long bones aged 25 years and over.

FIG. 6.—26 treated cases of osteosarcoma of other than long bones.

immediately consequent or within one month of completion of the course of irradiation (Fig. 3).

Comparable data of metastatic frequency for these three groups of patients are given in Table I.

TABLE I.—*Metastatic Spread of Osteosarcoma of Long Bones in Different Treatment Groups—Series A* (123 *Cases*)

| | Percentage with metastases (mean estimates) | | |
|---|---|---|---|
| | A1 amputation | A2 radical radiotherapy | A3 pre-operative radiotherapy |
| Month | (53) | (50) | (20) |
| 0 | 2 | 6 | 17 |
| 3 | 27 | 30 | 52 |
| 6 | 41 | 51 | 72 |
| 9 | 51 | 64 | 78 |
| 12 | 60 | 73 | 83 |
| 15 | 67 | 77 | 87 |
| 18 | 70 | 81 | 89 |
| 21 | 72 | 83 | 90 |
| 24 | 73 | 84 | 90 |
| 36 | 73 | 85 | 90 |
| 48 | 74 | 89 | 90 |
| 60 | 77 | 89 | 90 |

These mean estimates are derived from the mean disease-free curves of Fig. 1, 2 and 3.

*Series A*, of 123 cases, was also studied in respect of age irrespective of treatment:

Juveniles under 25 years—96 (Fig. 4) (classed as juveniles as the vast majority of this group had not yet attained skeletal maturity *i.e.* epiphyseal fusion and cessation of all bone growth).

Mature adults over 25 years—27 (Fig. 5). (This group implies skeletal maturity although periosteal bone growth may continue for some time.)

Table II shows the crude survival and mean disease-free rates for 123 osteosarcomata of long bones sub-divided into 68 tumours of femur, 36 of the tibia and fibula and 19 of the humerus.

*Series B*—All other sites (26).

Fig. 6 demonstrates the crude survival and DFI curves for these patients. The great majority were treated by radiotherapy; 2 patients, moribund when first seen, were excluded.

### RESULTS

*Series A. Long bone tumours* (Fig. 1, 2, 3)

In each group there were patients with overt metastases at the time of treatment. Both at 6 and 18 months the crude survival and mean DFI are better for the Group A1 treated by surgery alone. All three crude survival and mean DFI curves begin to level off at about 24 months, and at 60 months the best results are still shown by the patients treated by surgery only (A1). The differences between the groups, however, are not statistically significant. These cases extend over a period of 27 years (1946–72); thus in Group A2 14 patients were treated by the conventional 220/250 kV irradiation (until

TABLE II.—*Osteosarcoma of Long Bones—Series A, Comparative Results by Sites of Tumour*

| | Femur (68 cases) | | Tibia and Fibula (36 cases) | | Humerus (19 cases) | |
|---|---|---|---|---|---|---|
| Months from start of treatment | Mean disease-free (%) | Alive (%) | Mean disease-free (%) | Alive (%) | Mean disease-free (%) | Alive (%) |
| 0 | 92 | 100 | 97 | 100 | 95 | 100 |
| 3 | 68 | 94 | 77 | 97 | 66 | 94 |
| 6 | 48 | 79 | 56 | 89 | 43 | 89 |
| 9 | 42 | 62 | 41 | 75 | 34 | 58 |
| 12 | 34 | 55 | 31 | 61 | 29 | 53 |
| 15 | 31 | 47 | 28 | 47 | 22 | 33 |
| 18 | 27 | 45 | 24 | 39 | 20 | 28 |
| 21 | 24 | 38 | 23 | 26 | 17 | 28 |
| 24 | 23 | 37 | 23 | 26 | 17 | 22 |
| 30 | 20 | 31 | 23 | 23 | 17 | 22 |
| 36 | 20 | 27 | 23 | 23 | 17 | 22 |
| 48 | 18 | 21 | 23 | 23 | 17 | 17 |
| 60 | 15 | 18 | 21 | 21 | 17 | 17 |

1956), all others received radiocobalt or supervoltage therapy. The changing fashion in treatment over this period is shown in Fig. 7. In Group A3 16 patients received 220/250 kV radiotherapy.

*The effect of patients' age.—Long bones only* (Figs. 4, 5).—Throughout the duration of 36 months after treatment, both the crude survival and mean DFI are better for older patients (25 years). This was noted in a previous study (Price, 1966). All curves again tend to flatten out 24 months from the time of completing treatment. The differences between the 2 age groups are not statistically significant.

*The effect of sex.—Long bones only* (Table III).—Although the crude and mean disease-free survival rates are slightly better for females, the differences are not statistically significant.

*The effect of tumour site.—Long bones only.*—Reference to Table II shows no major differences in the mean disease-free

FIG. 7.—Trends in treatment of osteosarcoma of long bones.

TABLE III.—*Osteosarcoma of Long Bones—Series A, Comparative Results According to Sex*

| | 74 males | | 49 females | |
|---|---|---|---|---|
| Month | Mean disease-free (%) | Alive (%) | Mean disease-free (%) | Alive (%) |
| 0 | 94 | 100 | 94 | 100 |
| 3 | 65 | 99 | 78 | 98 |
| 6 | 48 | 81 | 52 | 88 |
| 9 | 40 | 60 | 43 | 73 |
| 12 | 30 | 51 | 36 | 65 |
| 15 | 28 | 43 | 31 | 49 |
| 18 | 24 | 40 | 26 | 40 |
| 21 | 21 | 32 | 24 | 35 |
| 24 | 20 | 31 | 24 | 33 |
| 30 | 18 | 24 | 24 | 33 |
| 36 | 18 | 23 | 24 | 28 |
| 48 | 17 | 17 | 22 | 27 |
| 60 | 16 | 16 | 16 | 23 |

rates for femoral tumours compared with those of tibia and fibula, and tumours of humerus, either at 24 or 60 months after treatment. The femoral cases showed a higher proportion of patients alive at 24 months, but at 60 months the site differences are small. Despite a general reluctance to amputate a tumour-bearing arm, the humerus cases did not fare markedly worse than patients with tumours of the leg.

Examination of tumour site distribution within the 3 different groups of long bone tumours therefore indicated no inherent bias due to these 2 factors after a period of 12 months.

The sex distribution also appeared to have no marked effect. Table IV shows the sex/age/site distribution of Groups A1, A2 and A3. The proportions of patients over 25 years of age in the 3 groups A1, A2 and A3 are respectively 28%, 18% and 15%. This preponderance of older persons in Group A1 may have contributed slightly to the better results observed for the cases treated by surgery only.

*Series B. Osteosarcoma of other sites* (26)

Data for this group are shown in Fig. 6. Three patients died of local spread of their

TABLE IV.—*Osteosarcoma Series A* (123 *cases*), *Sex, Age and Site Distribution of Tumours of Long Bones*

| Group | A1 Amputation | | | A2 Radical radiotherapy | | | A3 Pre-operative radiotherapy | | |
|---|---|---|---|---|---|---|---|---|---|
| | M | F | All | M | F | All | M | F | All |
| Sex | 33 | 20 | 53 | 27 | 23 | 50 | 14 | 6 | 20 |
| Age (years) | | | | | | | | | |
| 5–9 | 2 | 2 | 4 | 2 | 4 | 6 | — | — | — |
| 10–14 | 5 | 5 | 10 | 8 | 7 | 15 | 4 | 3 | 7 |
| 15–19 | 11 | 5 | 16 | 10 | 5 | 15 | 6 | 3 | 9 |
| 20–24 | 7 | 1 | 8 | 4 | 1 | 5 | 1 | — | 1 |
| 25–29 | 1 | — | 1 | 1 | 2 | 3 | — | — | — |
| 30–34 | — | 1 | 1 | 1 | 1 | 2 | — | — | — |
| 35–39 | 1 | 2 | 3 | — | 1 | 1 | 1 | — | 1 |
| 40–44 | 1 | — | 1 | — | 1 | 1 | — | — | — |
| 45–49 | 1 | 1 | 2 | — | — | — | 1 | — | 1 |
| 50–54 | 1 | — | 1 | — | — | — | — | — | — |
| 55–59 | 2 | 1 | 3 | 1 | — | 1 | 1 | — | 1 |
| 60–64 | 1 | — | 1 | — | — | — | — | — | — |
| 65–69 | — | 1 | 1 | — | — | — | — | — | — |
| 70–74 | — | — | — | — | 1 | 1 | — | — | — |
| 75–79 | — | — | — | — | — | — | — | — | — |
| 80– | — | 1 | 1 | — | — | — | — | — | — |
| Mean age | 24·4 | 26·7 | 25·2 | 18·0 | 19·7 | 18·7 | 22·6 | 15·5 | 20·5 |
| Sites | | | | | | | | | |
| Femur | 17 | 11 | 28 | 15 | 13 | 28 | 7 | 5 | 12 |
| Tibia | 11 | 9 | 20 | 6 | 3 | 9 | 3 | — | 3 |
| Humerus | 3 | — | 3 | 6 | 6 | 12 | 4 | — | 4 |
| Fibula | 2 | — | 2 | — | 1 | 1 | — | 1 | 1 |

tumour without evidence of metastatic spread (1 mandible, 2 ilium). The 2 patients not included in this group received no treatment for their tumour (1 calvarium, 1 vertebra), both dying from local spread within one month of their first being seen. The more rapid metastatic spread and shorter survival for this group may be noted from Fig. 6. At 5 years the crude survival rate and disease-free survivors were respectively 12% and 8%.

Table V gives the sex/age/site distribution for Series B; 69% of the patients of this group were over 25 years of age.

TABLE V.—*Osteosarcoma of Other than Long Bones—Series B* (26 *Cases*), *Sex, Age and Site Distribution*

| Group | Male | Female | All |
|---|---|---|---|
| Sex | 14 | 12 | 26 |
| Age (years) | | | |
| 5–9 | 1 | — | 1 |
| 10–14 | 1 | 1 | 2 |
| 15–19 | 2 | 1 | 3 |
| 20–24 | 1 | 1 | 2 |
| 25–29 | — | 1 | 1 |
| 30–34 | 3 | — | 3 |
| 35–39 | — | 2 | 2 |
| 40–44 | 1 | 1 | 2 |
| 45–49 | 1 | 1 | 2 |
| 50–54 | — | 1 | 1 |
| 55–59 | 2 | 1 | 3 |
| 60–64 | 1 | 2 | 3 |
| 65–69 | — | — | — |
| 70– | 1 | — | 1 |
| Mean age | 37·5 | 41·5 | 39·3 |
| Sites | | | |
| Mandible | 2 | 2 | 4 |
| Maxilla | 1 | — | 1 |
| Vertebrae | 1 | 1 | 2 |
| Ribs | 1 | 2 | 3 |
| Scapula | — | 1 | 1 |
| Sacrum | — | 2 | 2 |
| Sacro-iliac | — | 1 | 1 |
| Ilium | 7 | 2 | 9 |
| Ischium | 1 | — | 1 |
| Pubis | — | 1 | 1 |
| Scaphoid (foot) | 1 | — | 1 |

Of 26 patients, one was treated by amputation, 6 by resection and 19 by radiotherapy (11 by 200–220 kV, 5 by radiocobalt, 2 by supervoltage therapy and one by an unrecorded technique).

### DISCUSSION

Although crude survival statistics have been published by many authors for osteosarcoma, there are but few analytical studies of the known or estimated duration of the mean DFI and its converse, the rate of metastatic spread. Probably the diagrams published by Jenkin (1973) and Marcove *et al.* (1973) are those most closely comparable with the present study. Marcove *et al.* (1973) and Enneking and Marsh (1973) in particular emphasize the essential difficulties in constructing the DFI curves with anything approaching accuracy. In the absence of basic data in past records no statistical treatment can improve the value of such information.

Although in the Bristol series of long bone tumours the best results are shown in the surgically treated group, it is not suggested that these results provide any strong argument in favour of surgery as the preferred method of treatment. The pros and cons of this controversy are discussed at some length by Lee (1973) and other contributors in the Colston Papers, XXIV. Moreover, by combining results from many sources, Trifaud and Méary (1972) calculate crude 5 year survival rates of 21% for immediate surgery and 23·8% for radiotherapy with or without surgery. In this context there are many points to be considered and evaluated before deciding the mode of treatment.

The data given in this paper are presented for comparison with the results of new methods of treatment of osteosarcoma. For valid comparison the results over a 2 year period may be used, but in order to obtain the maximum information all new patients should be reviewed at monthly intervals after treatment with a radiograph of the chest in order to ascertain to the nearest month the actual duration of the DFI. This should be done for 24 months. As seen from all the curves given, from that time onwards the prognosis improves and bi- or tri-monthly re-examination may suffice up to the 5 year interval. This regimen not only allows an accurate estimate of the actual

FIG. 8.—Osteosarcoma of long bones amputated without radiotherapy. 95% confidence limits for disease-free curve.

DFI but also provides early information of metastatic spread, for which active treatment should be instituted. Obviously, great caution is required in the interpretation of data derived from small groups of cases. Fig. 8 indicates the 95% confidence limits from small groups of 5, 10 and 25 cases estimated from the observed data according to the formula—$p \pm 2$ times the standard error which is calculated as

$$\sqrt{\frac{p \times q}{n}}$$

where $p$ is the percentage disease-free, $q = 100 - p$, and $n$ is the number of cases. (Bradford Hill, 1971). To achieve a meaningful result in 3 years, it would be necessary to collect records of at least 25 patients, consisting of all available long bone osteosarcoma cases in one year from a population of about 15 million persons (Fig. 8).

Any future progress in the treatment of osteosarcoma can be anticipated only if it is realized that *probably all patients when first seen have micro-metastases.* With this practical problem in mind, a strong plea is made for the organization of a collaborative treatment group of clinicians, supported by radiodiagnosticians and pathologists, to study and compare the prophylaxis and treatment of osteosarcoma by promising new methods, which may include prophylactic lung irradiation and chemotherapy, in the programme of management.

The authors are indebted to the numerous clinicians and other colleagues who by referring their cases to the Bone Tumour Registry have provided this series of osteosarcomata collected during the past 27 years. Their sustained interest and help in this study have been invaluable, together with the advice given by Miss E. H. L. Duncan, Medical Statistician of the University of Bristol Department of Public Health. Thanks are also due to Mrs J. E. Nutt for much help in collecting and maintaining case records and for clerical assistance.

The Bristol Bone Tumour Registry is supported by generous grants from the Cancer Campaign for Research.

### REFERENCES

CORTES, E. P., HOLLAND, J. F., WANG, J. J. & SINKS, L. F. (1973) Chemotherapy of Advanced Osteosarcoma. In *The Colston Papers* XXIV. Ed. C. H. G. Price and F. G. M. Ross. London: Butterworth, p. 265.

Editorial Annotation (1971) *British Medical Journal*, ii, 355.

Enneking, W. F. & Marsh, B. (1973) Immunologic Aspects of Osteosarcoma. In *The Colston Papers* XXIV. Ed. C. H. G. Price and F. G. M. Ross. London: Butterworth. p. 431.

Hill, A. Bradford (1971) *Principles of Medical Statistics*, 9th Ed. London: The Lancet Ltd. p. 131.

Jaffe, N. (1972) Recent Advances in the Chemotherapy of Metastatic Osteogenic Sarcoma. *Cancer, N.Y.*, **30**, 1627.

Jenkin, R. D. T. (1973) The Management of Osteo-Sarcoma and Ewing's Sarcoma. In *The Colston Papers* XXIV. Ed. C. H. G. Price and F. G. M. Ross. London: Butterworth. p. 229.

Kuehn, P. G., Tamoney, H. J. & Gossling, H. R. (1970) Iliac vein Occlusion prior to Amputation for Sarcoma. *Cancer, N.Y.*, **26**, 536.

Lee, E. Stanley (1973) The Problems of Biopsy in the Westminster Hospital Experience. In *The Colston Papers* XXIV. Ed. C. H. G. Price and F. G. M. Ross. London: Butterworth. p. 163.

Marcove, R. C., Miké, V., Hajek, J. V., Levin, A. G. & Hutter, R. V. P. (1970) Osteogenic Sarcoma under the age of Twenty-one. *J. Bone Jt. Surg.*, **52-A**, 411.

Marcove, R. C., Southam, C. M., Levin, A. G., Huvos, A. G. & Miké, V. (1973) New Trends in the Treatment of Osteogenic Sarcoma. In *The Colston Papers* XXIV. Ed. C. H. G. Price and F. G. M. Ross. London: Butterworth. p. 313.

Martini, N., Huvos, A. G., Miké, V., Marcove, R. C. & Beatty, E. J. Jr (1971) Multiple Pulmonary Resections in the Treatment of Osteogenic Sarcoma. *Ann. Thorac. Surg.*, **12**, 271.

Newton, K. A. (1973) Prophylactic Irradiation of the Lung in Bone Sarcoma. In *The Colston Papers* XXIV. Ed. C. H. G. Price and F. G. M. Ross. London: Butterworth. p. 307.

Ohno, T. (1971) Bronchial Artery Infusion with Anti-cancer Agents in the Treatment of Osteosarcoma. *Cancer, N.Y.*, **27**, 549.

Owen, L. N. (1973) Transplantation of Canine Osteosarcoma. In *The Colston Papers* XXIV. Ed. C. H. G. Price and F. G. M. Ross. London: Butterworth. p. 327.

Price, C. H. G. (1966) The Prognosis of Osteosarcoma. *Br. J. Radiol.*, **39**, 181.

Price, C. H. G. & Goldie, W. (1969) Paget's Sarcoma in Bone. *J. Bone Jt. Surg.*, **51-B**, 205.

Rosen, G., Tan, C., Suwansirikul, S. & Murphy, M. L. (1973) *Proc. Amer. Ass. Cancer Res.*, **14**, 95.

Trifaud, A. & Méary, R. (1972) *Prognostic et Traitement des Sarcomes Ostéogéniques*. Paris: Masson. p. 123.