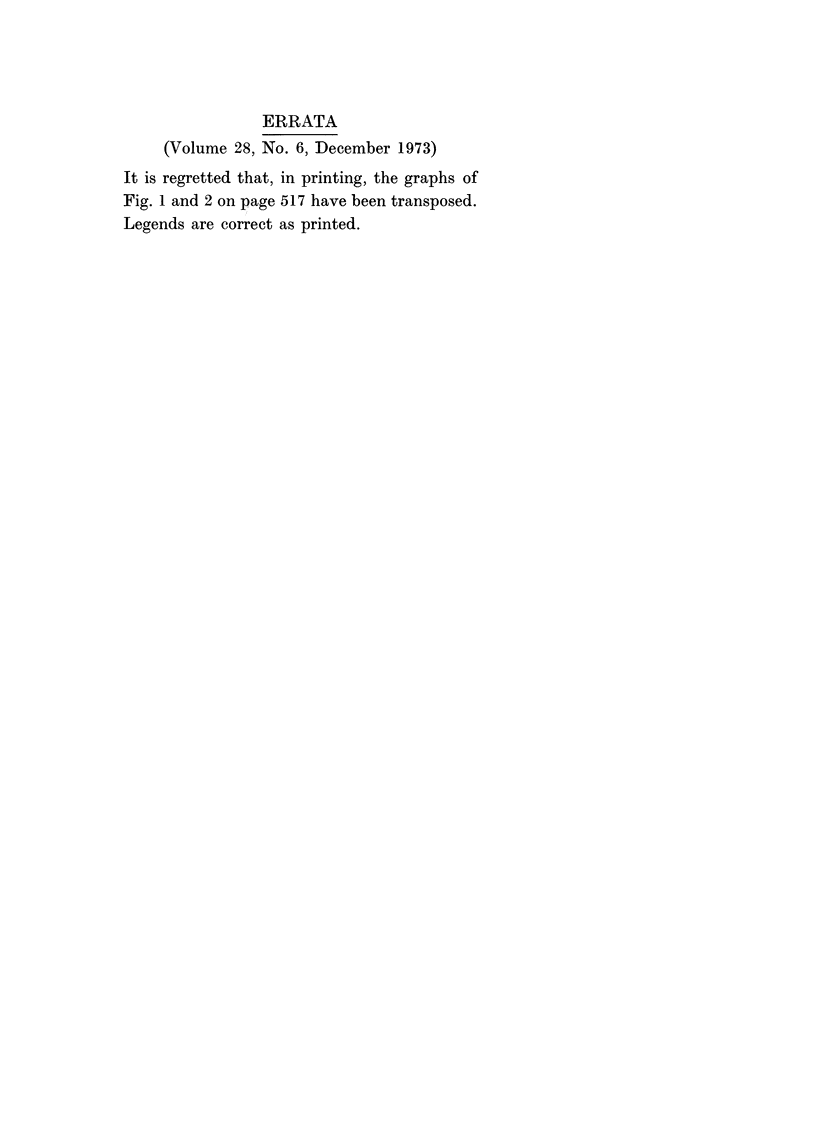# Errata

**Published:** 1974-01

**Authors:** 


### ERRATA

(Volume 28, No. 6, December 1973)

It is regretted that, in printing, the graphs of Fig. 1 and 2 on page 517 have been transposed. Legends are correct as printed.